# Tracking hematopoietic precursor division ex vivo in real time

**DOI:** 10.1186/s13287-017-0767-z

**Published:** 2018-01-23

**Authors:** Yuchen Wang, Hong Tian, Wenzhi Cai, Zhaorui Lian, Dheeraj Bhavanasi, Chao Wu, Tomohiko Sato, Mineo Kurokawa, Depei Wu, Li Fu, Hong Wang, Hao Shen, Dong Liang, Jian Huang

**Affiliations:** 1 Department of Physiology & Pathophysiology, School of Basic Medical Science, Peking University, Beijing, People’s Republic of China; 2 Department of Pathology and Laboratory Medicine, Lewis Katz School of Medicine, Temple University, Philadelphia, PA 19140 USA; 3 Jiangsu Institute of Hematology, The First Affiliated Hospital of Soochow University, Suzhou, People’s Republic of China; 4 Department of Medicine (Hematology-Oncology), University of Pennsylvania School of Medicine, Philadelphia, Pennsylvania USA; 5 Fox Chase Cancer Center, Philadelphia, Pennsylvania USA; 6 Department of Hematology and Oncology, University of Tokyo, Bunkyo-ku, Tokyo 113-8655 Japan; 7 Department of Physiology and Pathophysiology, School of Basic Medical Science, Tianjin Medical University, Tianjin, 300070 People’s Republic of China; 8 Center for Metabolic Disease Research, Department of Pharmacology, Lewis Katz School of Medicine, Temple University, Philadelphia, USA; 9 Department of Microbiology, University of Pennsylvania School of Medicine, Philadelphia, Pennsylvania USA; 10 Department of Prenatal Diagnosis, Obstetrics and Gynecology Hospital Affiliated to Nanjing Medical University, Nanjing, 210004 Jiangsu People’s Republic of China

**Keywords:** Hematopoietic stem cell-cell division, Time lapse tracking-leukemogenesis

## Abstract

**Background:**

Deciphering molecular mechanisms underlying the division of hematopoietic stem cells (HSCs) and malignant precursors would improve our understanding of the basis of stem cell-fate decisions and oncogenic transformation.

**Methods:**

Using a novel reporter of hematopoietic precursor, Evi1-GFP, we tracked the division of hematopoietic precursors in culture in real time.

**Results:**

First, we confirmed that Evi1-GFP is a faithful reporter of HSC activity and identified three dividing patterns of HSCs: symmetric renewal, symmetric differentiation, and asymmetric division. Moreover, we found that the cytokine and growth factor combination (STIF) promotes symmetric renewal, whereas OP9 stromal cells balance symmetric renewal and differentiation of HSCs ex vivo. Interestingly, we found that *Tet2* knockout HSCs underwent more symmetric differentiation in culture compared with the wild-type control. Intriguingly, OP9 stromal cells reverse the phenotype of *Tet2* knockout HSCs ex vivo. Furthermore, we demonstrated that *Tet2*^*–/–*^;*Flt3*^ITD^ acute myeloid leukemia (AML) precursors primarily underwent symmetric renewal divisions in culture. Mechanistically, we demonstrated that inhibiting DNA methylation can reverse the aberrant division phenotypes of *Tet2*^*–/–*^ and *Tet2*^*–/–*^;*FLT3*^ITD^ precursors, suggesting that abnormal DNA methylation plays an important role in controlling (pre-)leukemic precursor fate decision ex vivo.

**Conclusions:**

Our study exploited a new system to explore the molecular mechanisms of the regulation of benign and malignant hematopoietic precursor division ex vivo. The knowledge learned from these studies will provide new insights into the molecular mechanisms of HSC fate decision and leukemogenesis.

**Electronic supplementary material:**

The online version of this article (10.1186/s13287-017-0767-z) contains supplementary material, which is available to authorized users.

## Background

Hematopoietic stem cells (HSCs) are characterized by their self-renewal potential and ability to differentiate into multiple blood lineages. Several decades of successful HSC transplantations have demonstrated the therapeutic importance of HSCs [1, 2]. Stem cells can undergo asymmetric and symmetric divisions, which lead to self-renewal and differentiation, but the key factors that affect this decision of HSCs remain unknown. During asymmetric division, one daughter cell remains a stem cell, while the other becomes committed. In contrast, during symmetric divisions, a stem cell give rises to two stem cells (symmetric renewal) or two committed cells (symmetric commitment). When stem cells divide asymmetrically, tissues maintain homeostasis of stem cells while allowing a progressive increase in the number of differentiated cells. Conversely, symmetric renewal divisions result in expansion of the immature stem or precursor pool, whereas symmetric commitment divisions allow only differentiated cells to be generated [3, 4].

Ecotropic viral integration site 1 (EVI1) is an oncogenic transcription factor that belongs to the SET/PR domain protein family. The *Evi1* locus was initially discovered as a common target of retroviral integration site in murine myeloid leukemias [5, 6]. *Evi1*-deficient embryos show a marked reduction in hematopoietic stem/progenitor cells (HSPC) in the para-aortic splanchnopleura (P-Sp) region, as well as losing their long-term repopulating capacity in vivo. In addition, HSCs in *Evi1*-null fetal livers are significantly reduced in number with defective multilineage reconstitution ability. Conditional deletion of *Evi1* in adult mice leads to a profound loss of HSC self-renewal activity, but does not affect blood cell lineage commitment [5, 6]. These findings suggest that EVI1 is essential for HSC self-renewal in the fetal and adult hematopoietic system. In an elegant study of EVI1 in HSCs with a newly constructed Evi1-green fluorescent protein (GFP) reporter mouse line, Kataoka et al. demonstrated that EVI1 is expressed exclusively in the HSC population in the bone marrow, and its expression marks hematopoietic cells with long-term multilineage repopulating activity [7]. Of note, the GFP knock-in to *Evi1* locus does not perturb the function of *Evi1* in the hematopoiesis system [7].

Ex vivo expansion of functional long-term HSCs (LT-HSC) has been a challenging goal since it is not clear what intrinsic and extrinsic signals are required to control the proliferation of HSCs [8, 9]. Several publications suggest that stem cell factor (SCF), thrombopoietin (TPO), and FMS-like tyrosine kinase-3 ligand (Flt3-L) are essential for HSCs in culture [10]. Lodish and colleagues identified STIF (SCF + TPO + insulin-like growth factor (IGF)-2 + fibroblast growth factor (FGF)-1) as a HSC amplification recipe, and that this cocktail can expand mouse and human LT-HSC ex-vivo culture [11, 12]. OP9 stromal cells are derived from op/op transgenic mice that genetically lack macrophage-colony stimulating factor (M-CSF). OP9 cells can support differentiation of embryonic stem cells to hematopoietic cells as well as maintaining HSC fate in vitro [13]. A previous study demonstrated that OP9 stromal cells direct HSCs to undergo more symmetric renewal divisions than 7 F2 stromal cells, an osteoblastic cell line isolated from p53^−/−^ mice [14].

Numerous studies have demonstrated that epigenetic regulators play a critical role in HSC function, especially DNA methylation [15–18]. DNA methylation patterns, typically methylated CpGs, are established during early development. DNA methyltransferase enzymes (DNMTs) are responsible for both establishment and maintenance of these modifications throughout life. DNA demethylation is the process of removal of a methyl group from nucleotides in DNA. The ten-eleven translocation (TET) proteins TET1, TET2, and TET3 were identified as a family of cytosine dioxygenases; they are capable of converting 5-methylcytosine (5mC) to 5-hydroxymethylcytosine (5hmC) and its derivatives, 5-formylcytosine and 5-carboxylcytosine [19–21]. TET-mediated DNA demethylation has been demonstrated as one mechanism for reactivation of genes that have been transcriptionally silenced by 5mC. It has also been speculated that 5hmC may function as a unique DNA modification that imparts distinct epigenetic information on the underlying genome in some contexts. The genes regulating active DNA demethylation, the TET family of enzymes, are also important for HSC function [22]. Loss of expression of *Tet2* in HSCs causes an increased primitive compartment including both stem and progenitor cells, suggesting that HSCs deficient in *Tet2* promote HSC self-renewal in vivo [23–25]. Recently, it was reported that loss of *Tet2* together with *Flt3*^ITD^ induces acute myeloid leukemia (AML) in a mouse model. Of note, this AML model has a defined leukemia stem cell (LSC) population with a characteristic transcriptional and epigenetic profile [26].

To explore the intrinsic and extrinsic signals that control HSC self-renewal and differentiation, we utilized transgenic Evi1 reporter (Evi1-GFP) mice [7], in which GFP fluorescence marks HSC activity, to harvest hematopoietic precursors. Using GFP^+^ HSCs from the Evi1-GFP mice, we have employed time-lapse microscopy to track hematopoietic precursor division in real time ex vivo. We also defined whether the rate of division and the pattern changes in the context of different treatments. We found that the STIF combination can dramatically promote symmetric renewal whereas OP9 stromal cells balance the renewal and differentiation of hematopoietic precursors ex vivo. We also found that the balance of symmetric and asymmetric division can be subverted by *Tet2* knockout and restored by OP9 stromal cells, suggesting that both extrinsic and intrinsic cues influence HSC division in culture. Furthermore, we demonstrated that *Tet2*^–/–^*;Flt3*^ITD^ LSCs underwent more symmetric renewal ex vivo, consistent with the concept that oncogenes promote the renewal of hematopoietic precursors [14, 27]. Our study exploited a novel system to trace the division patterns of hematopoietic precursor cells ex vivo in real time, which can be employed to investigate the intrinsic and extrinsic signaling regulation of the fate of benign and malignant hematopoietic precursors in culture.

## Methods

### Mice

C57BL/6 wild-type (CD45.2^+^) mice were from the Jackson Laboratory. Evi1-GFP transgenic mice were generously provided by Dr. Mineo Kurokawa at the University of Tokyo [7] while *Tet2* knockout (023359) and *Flt3*^ITD^ mice (011112) were obtained from the Jackson Laboratory [23, 28]. Mice were genotyped in-house by polymerase chain reaction (PCR). For all time-lapse imaging experiments, we used Evi1-GFP homozygous mice to detect stronger GFP signal than heterozygous mice. All mice were bred in-house in a pathogen-free mouse facility at the Temple University Lewis Katz School of Medicine. Animal experiments were performed in accordance with guidelines approved by the Institutional Animal Care and Use Committee (IACUC) at Temple University.

### Culture medium

The base medium used was Gibco FluoroBrite™ Dulbecco’s modified Eagle’s medium (DMEM; Life Technologies A18967) supplemented with 5% fetal bovine serum (FBS), 0.01% bovine serum albumin (BSA), 10 μg/ml transferrin, 25 μg/ml insulin, 0.1 mM β-ME, 2 mM l-glutamine, 50 IU/ml penicillin/streptomycin, 20 ng/ml mouse SCF, 20 ng/ml mouse TPO, 20 ng/ml FLT3-L. STIF medium used was Gibco FluoroBrite™ DMEM medium supplemented with 10 g/mL heparin (Sigma-Aldrich, St Louis, MO), 10 ng/mL mouse SCF, 20 ng/mL mouse TPO, 20 ng/mL mouse IGF-2 (all from R&D Systems), and 10 ng/mL human FGF-1 (Invitrogen, Frederick, MD).

### Fluorescence-activated cell sorting and isolation of HSCs

Bone marrow cells were flushed from the long bones (tibias and femurs) of mice with phosphate-buffered solution (PBS) without calcium or magnesium [29, 30]. For detection of LSK cells, whole bone marrow cells were incubated with phycoerythrin (PE) anti-mouse Lineage Cocktail antibody, Brilliant Violet 421™-conjugated antibody to Sca1 (D7), and allophycocyanin (APC)-conjugated antibody to c-Kit (ACK2). For detection of SLAM markers, PE Cy5-conjugated antibody to CD48 (HM48-1) and PE Cy7-conjugated antibody to CD150 (TC15-12 F 12.2) were used. All antibodies were purchased from Biolegend except for antibodies to c-Kit, which were purchased from eBioscience. Antibodies to lineage, Sca1, and c-Kit were diluted 1:100. Nonviable cells were excluded using the viability dye 7-AAD (50 μg/ml). Cells were sorted with a FACSAria (Becton Dickinson) automated cell sorter. Analysis was performed on FACSCanto flow cytometer (Becton Dickinson). Data were analyzed using FlowJo software (Tree Star).

### In vitro methylcellulose assays

GFP^+^ or GFP^–^ LSK cells from Evi1-GFP mice were sorted and cultured in complete methylcellulose medium (Methocult GF M3434 from StemCell Technologies). Colonies were scored after 7 days of culture and identified by morphology.

### In vivo analysis of HSC function

GFP^+^ or GFP^–^ LSK cells from Evi1-GFP mice were sorted and 100, 500, or 2500 GFP^+^ or GFP^–^ cells were subsequently transplanted into groups of SJL (CD45.1^+^) congenic mice hosts (three mice/dose). Host mice were lethally irradiated using a Cs-137 Irradiator in two equal doses of 500 rads separated by at least 2 h. Cells were injected into the retro-orbital venous sinus of anesthetized recipient mice. All transplanted hosts are subsequently maintained on antibiotic water. Beginning 4 weeks after transplantation and continuing for at least 16 weeks, peripheral blood was collected from the tail veins of recipient mice and analyzed by FCM for the lineage markers B220 (6B2), Mac-1 (M1/70), CD4 (L3T4) and CD8 (Ly-3), and Gr-1 (8C5) to monitor engraftment. Donor and host cells were distinguished by expression of CD45.1 (A20, eBioscience) and CD45.2 (104, eBioscience) to determine the level of chimerism.

### Time-lapse imaging

GFP^+^ LSK cells from Evi1-GFP mice were sorted and cultured on glass bottomed 35-mm petri dishes. Unattended time-lapse movies chose random GFP^+^ hematopoietic precursors sorted with minimal intensity fluorescent excitation, provided by camera-triggered and Uniblitz-shuttered illumination. Deltavision Softworx software was used for acquisition and processing. The blank and GFP^+^ fields were both detected at the same time. For tracking the movies steadily and continuously, a Photometrics CoolSnap HQ high-resolution CCD camera was used. The environmental chamber surrounding the whole microscope was used for temperature and CO_2_ control. Over a period of 24 h, cells were maintained at 37 °C, 5% CO_2_, and the humidification level was saturated. A 40× objective was used for tracking GFP^+^ cells in 15 non-overlapping wide fields to aid in cell identification. Targeted cells were observed in movie replay and analyzed by Fiji software. Criteria for scoring division patterns were set as described previously [14].

### Statistical analysis

Statistical analysis was performed by unpaired Student’s *t* test. GraphPad Prism was used for statistical analysis.

## Results

### Evi1-GFP is a faithful reporter of HSC activity

**Fig. 1:**
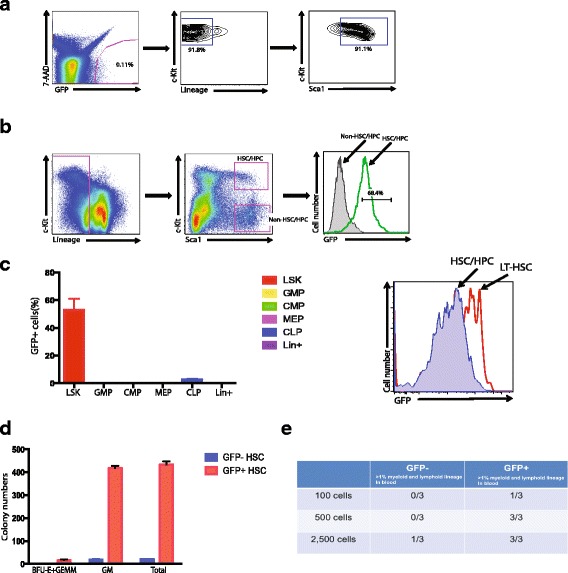
Evi1-GFP is a faithful reporter for hematopoietic precursors. **a** Representative data from FACS analysis of green fluorescent protein (GFP)^+^ cells of Evi1-GFP mouse. The GFP^+^ population was further analyzed by staining lineage, Sca1, and c-Kit markers. **b** Representative data from FACS analysis of bone marrow stem and progenitor populations of Evi1-GFP mouse. The GFP^+^ populations from Lin-Sca1^+^c-Kit^+^ (LSK) and Lin-Sca1^+^c-Kit^–^ are compared. **c** (left) The quantitative analysis from FACS analysis of Evi1-GFP mouse bone marrow. The percentage of GFP^+^ cells in LSK, GMP, CMP, CLP, and lineage positive populations is shown. (right) Representative data from FACS analysis of bone marrow stem and progenitor populations of Evi1-GFP mouse. The GFP^+^ populations from Lin-Sca1^+^c-Kit^+^ and Lin-Sca1^+^c-Kit^+^CD150^+^CD48^–^ are compared. **d** Colony formation assay (CFC) was performed with sorted GFP^+^ cells from Evi1-GFP mouse. The number of BFU-E + GEMM (erythroid, granulocyte, erythrocyte, monocyte, megakaryocyte), GM (granulocyte, monocyte), and total colony number is shown. **e** The limiting dilution assay was performed to measure the number of hematopoietic stem cells (HSCs) in the GFP^+^ population of Evi1-GFP mouse; 100, 500, and 2500 GFP^+^ cells from Evi1-GFP mouse were sorted and transplanted into lethally irradiated mice in combination with 3 × 10^5^ recipient-derived bone marrow (BM) cells. The percentage of donor-derived cells in BM was analyzed 16 weeks after reconstitution. Chimerism greater than 1% donor-derived cells was considered positive engraftment. Values show the number of mice with positive engraftment/total mice transplanted in GFP^–^ and GFP^+^ groups

To explore the mechanisms by which HSCs regulate symmetric and asymmetric division, we employed a green fluorescent protein (GFP) knock-in for the *Evi1* gene in mice. With this newly constructed Evi1-GFP reporter mouse line, Kataoka et al. demonstrated that EVI1 is expressed exclusively in the HSC population in the bone marrow, and its expression marks hematopoietic cells with long-term multilineage repopulating activity [7]. Consistent with their data, we found that the GFP signal correlates well with phenotypic HSCs (LSK population) in Evi1-GFP mouse bone marrow. Specifically, 91.1% of GFP^+^ cells from Evi1-GFP mice are phenotypic HSCs (LSK population) and, conversely, 68.4% of LSK cells are GFP^+^ (Fig. 1a, b). Of note, EVI1 is expressed exclusively in the phenotypic HSC population, but not progenitors and lineage committed cells, in the bone marrow (Fig. 1c, left). Furthermore, higher GFP intensity was detected within the LSK fraction using SLAM family receptors (CD48 and CD150) than whole LSK population (HSC/HPC) (Fig. 1c, right), suggesting that the GFP intensity correlates with HSC property in Evi1-GFP mouse bone marrow. As a functional readout, we found that GFP^+^ cells from Evi1-GFP mice formed 20-fold more colonies than GFP^–^ cells in colony formation assays (Fig. 1d). Most importantly, in the gold standard bone marrow transplantation assay (limiting dilution assay), only GFP^+^ cells from Evi1-GFP mice can repopulate lethally irradiated recipients (Fig. 1e). Collectively, our data indicate that GFP is indeed a faithful reporter of HSC activity in Evi1-GFP mouse bone marrow. Thus, the GFP signal can serve as a surrogate of HSC activity in culture, allowing us to track HSC activity ex vivo.

### Hematopoietic precursors undergo symmetric and asymmetric division

**Fig. 2:**
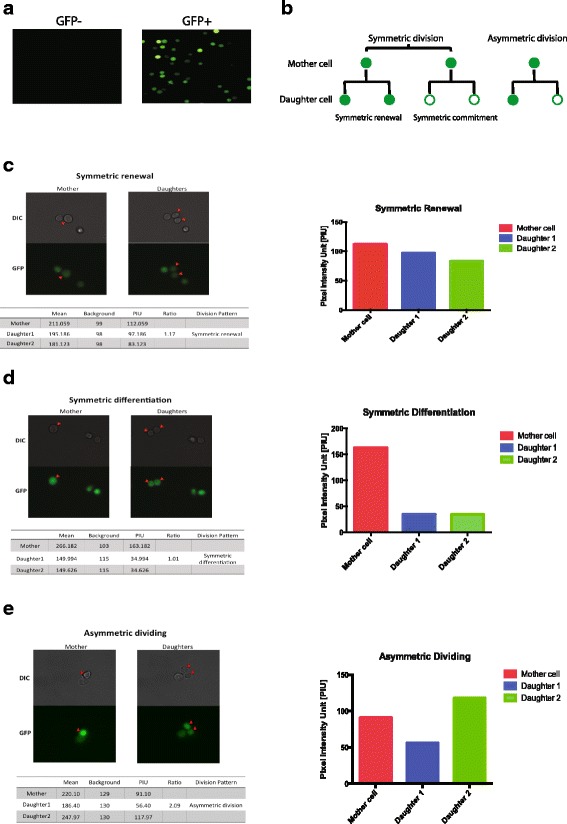
GFP^+^ precursors from Evi1-GFP mouse underwent both symmetric and asymmetric division ex vivo*.* **a** Representative microscopic data are shown for green fluorescent protein (GFP)^+^ and GFP^–^ cells sorted from Evi1-GFP mouse bone marrow. **b** The three dividing patterns of GFP^+^ hematopoietic precursors. When GFP^+^ precursors divide, they give rise to two GFP^−^ daughter cells (symmetric commitment), two GFP^+^ daughter cells (symmetric renewal), or one GFP^−^ and one GFP^+^ daughter cell (asymmetric division). **c** Representative microscopic data of symmetric renewal division of GFP precursors in culture. The GFP pixel intensity unit (PIU) and the ratio was calculated and is shown. The quantification of PIU of mother and daughters is shown on the right. **d** Representative microscopic data of symmetric differentiation division of GFP precursors in culture. The GFP PIU and the ratio was calculated and is shown. The quantification of PIU of mother and daughters is shown on the right. **e** Representative microscopic data of asymmetric division of GFP precursors in culture. The GFP PIU and the ratio was calculated and is shown. The quantification of PIU of mother and daughters is shown on the right

To identify the division pattern of hematopoietic stem/progenitor cells, we sorted Evi1-GFP^+^ cells from Evi1-GFP mouse bone marrow, plated them in glass-bottomed petri dishes, and tracked cell divisions using time-lapse inverted microscopy. Multiple GFP^+^ cells in the dish were identified, recorded, and revisited every 10 min over a period of 24 h. To track GFP^+^ division ex vivo, we visualized the cells in both DIC and GFP and were able to detect the very bright GFP signal of hematopoietic precursors in culture (Fig. 2a). Next, we tracked multiple GFP^+^ hematopoietic precursors dividing in culture and calculated the GFP pixel intensity unit (PIU) of the mother and daughter cells to determine the division pattern. It is well known that hematopoietic precursors are able to undergo three types of divisions: symmetric renewal, symmetric commitment, and asymmetric division (Fig. 2b). Based on the literature [14], we set the PIU ratio to 1.5-fold as the threshold for determining whether the mother cells gave rise to two GFP^+^ daughter cells (symmetric renewal), two GFP^–^ daughter cells (symmetric commitment), or one GFP^–^ and one GFP^+^ daughter cell (asymmetric division).

Consistent with the literature [14, 31, 32], we found that hematopoietic precursors display all three types of division ex vivo in our system. Specifically, we observed that a proportion of GFP^+^ cells underwent symmetric renewal and they were dividing and maintaining equivalent levels of GFP in both daughters (Fig. 2c and Additional file 1: Movie S1). There were also GFP^+^ cells that underwent symmetric commitment and the cells were dividing and downregulating GFP in both daughter cells (Fig. 2d and Additional file 2: Movie S2). Moreover, there was a small percentage of GFP^+^ cells undergoing asymmetric division and the cells were dividing and downregulating GFP in only one daughter (Fig. 2e and Additional file 3: Movie S3). Taken together, these data indicate that hematopoietic precursors can undergo both symmetric and asymmetric divisions in our system.

**Fig. 3:**
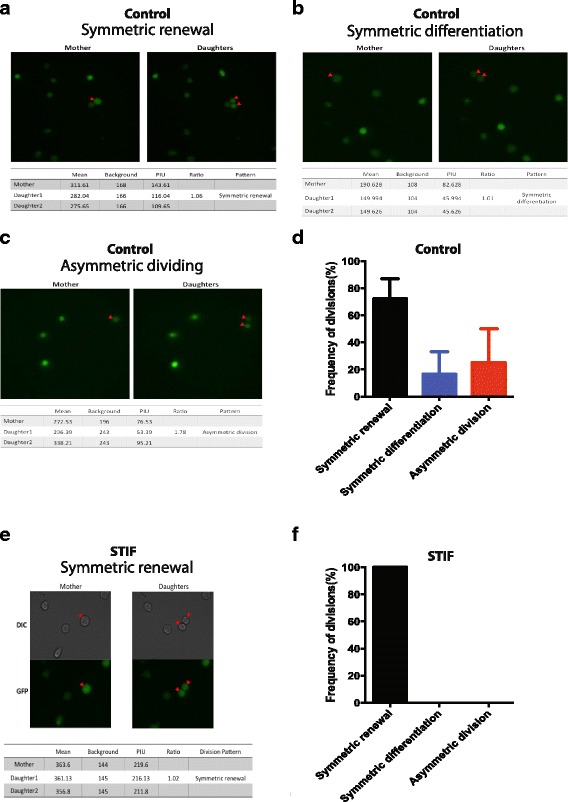
Cytokines/growth factors affect the division pattern of hematopoietic precursors. **a**–**c** Representative microscopic data of symmetric renewal, symmetric differentiation, and asymmetric division of green fluorescent protein (GFP)^+^ hematopoietic precursors harvested from Evi1-GFP mouse. The GFP pixel intensity unit (PIU) and the ratio was calculated and is shown. **d** The summary of division patterns of Evi1-GFP^+^ precursors from three independent experiments. The frequency of symmetric self-renewal, symmetric differentiation, and asymmetric division of GFP^+^ precursors under base conditions is shown. Error bars represent standard error of the mean (SEM). **e** Representative microscopic data are shown for GFP^+^ cells treated with STIF combinations. The GFP PIU and the ratio was calculated and is shown. **f** The frequency of symmetric self-renewal, symmetric differentiation, and asymmetric division of GFP^+^ treated with STIF (SCF + TPO + IGF-2 + FGF-1) combinations is shown. The data are a summary from three independent experiments. Error bars represent SEM

We next quantitatively analyzed the pattern of hematopoietic precursors dividing under several different conditions (different concentrations of cytokines and serum). The percentage of GFP^+^ cells dividing over 24 h varied from 0% to 14.8% (data not shown), suggesting that culture conditions are critical in controlling the division of HSCs. As an optimal culture condition, we chose SCF 20 ng/ml, TPO 20 ng/ml, and FLT3-L 20 ng/ml with 5% serum as our base culture medium. Under this condition, the percentage of GFP^+^ cells dividing over 24 h was 12.2 ± 2.3% from three independent experiments. Importantly, the GFP^+^ cells underwent symmetric renewal (69.2%), symmetric differentiation (15.4%), and asymmetric division (15.4%) under this condition (Fig. 3a–d).

### The cytokines, growth factors, and stromal cells influence the balance of hematopoietic precursor division

Previous studies demonstrated that cytokine and growth factor combinations can expand functional HSCs in culture [8, 11, 33]. To test whether cytokines and growth factors can affect the balance of symmetric and asymmetric division ex vivo, we treated Evi1-GFP^+^ cells with STIF (SCF, TPO, IGF-2, and FGF-1) and tracked GFP^+^ cells dividing for 24 h. Previously, STIF has been shown to dramatically expand HSCs ex vivo [11]. Consistent with the published data, we found that 100% of STIF-treated GFP^+^ hematopoietic precursors underwent symmetric renewal (Fig. 3e, f), suggesting that the precursors were expanded under this culture condition.

**Fig. 4:**
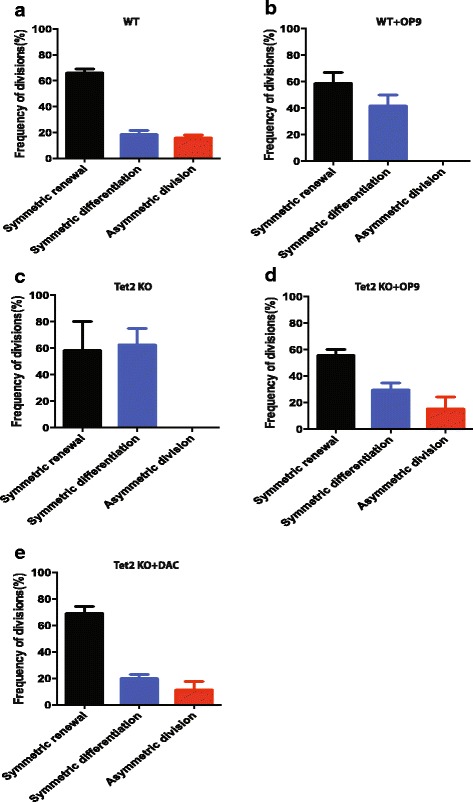
*Tet2* knockout hematopoietic precursors show distinct division pattern. **a** The frequency of symmetric self-renewal, symmetric differentiation, and asymmetric division of GFP^+^ precursors from wild-type (WT) mouse is shown. The data are a summary from three independent experiments. Error bars represent standard error of the mean (SEM). **b** The frequency of symmetric self-renewal, symmetric differentiation, and asymmetric division of wild-type GFP^+^ co-cultured with OP9 stromal cells is shown. OP9 stromal cells were plated in the culture dish 24 h before the time-lapse experiment. Then GFP^+^ hematopoietic precursors were sorted and plated on OP9 stromal cells for tracking of the division of HSCs. The data are a summary from three independent experiments. Error bars represent SEM. **c** The frequency of symmetric self-renewal, symmetric differentiation, and asymmetric division of *Tet2* knockout (KO) GFP^+^ cells is shown. The data are a summary from three independent experiments. Error bars represent SEM. **d** The frequency of symmetric self-renewal, symmetric differentiation, and asymmetric division of *Tet2* knockout GFP^+^ cells co-cultured with OP9 stromal cells is shown. OP9 stromal cells were plated in the culture dish 24 h before the time-lapse experiment. Then GFP^+^ hematopoietic precursors were sorted and plated on OP9 stromal cells for tracking of the division of HSCs. The data are a summary from three independent experiments. Error bars represent SEM. **e** The frequency of symmetric self-renewal, symmetric differentiation, and asymmetric division of *Tet2* knockout GFP^+^ cells treated with 5-aza-2′-deoxycytidine (DAC) 500 nM is shown. The data are a summary from three independent experiments. Error bars represent SEM

Previous study has shown that stromal cells can influence the pattern of hematopoietic precursors division [14]. The op/op transgenic mice-derived OP9 stromal cells directed HSCs to more symmetric renewal divisions than did 7 F2 stromal cells which were derived from p53^−/−^ osteoblastic cells [14]. Intriguingly, we found that OP9 stromal cells promote HSCs/HPCs to symmetric renewal divisions with 60% of precursors undergoing symmetric renewal divisions, whereas 40% of the HSCs underwent symmetric commitment (Fig. 4a, b), suggesting that culture microenvironments can influence the balance of symmetric and asymmetric division of HSCs. Furthermore, we found that either irradiated OP9 or OP9 supernatant was able to change the dividing pattern of precursors ex vivo (Additional file 4: Figure S1a, b), suggesting that both the interaction between OP9 and HSCs and paracrine secretion from OP9 are important to direct the fate decision of precursors. Hence, future research should aim to decipher the roles of Notch and/or Wnt signaling in regulating the division of precursors in culture. Collectively, we have exploited a new reporter system for investigating the extrinsic and intrinsic factors which can influence the division pattern of hematopoietic precursors in culture.

### *Tet2* knockout leads to more symmetric differentiation ex vivo

Accumulating evidence has shown that TET2 plays a key role in regulating HSC function. Loss of *Tet2* can increase the self-renewal of hematopoietic precursors in vivo. Specifically, *Tet2* loss leads to a progressive enlargement of the hematopoietic stem cell compartment and eventual myeloproliferation in vivo [25]. How TET2 affects hematopoietic precursor division in culture has not been studied. To address this question, we crossed *Tet2*^–/–^ mouse to Evi1-GFP mouse, harvested GFP^+^ cells, and examined the dividing pattern of *Tet2* null hematopoietic precursors ex vivo. First, we found that the *Tet2* knockout mouse has expanded phenotypic (LSK) hematopoietic precursor compartment by flow cytometry analysis as reported previously (Additional file 5: Figure S2a). Interestingly, the *Tet2*^–/–^;Evi1-GFP mouse maintains comparable frequency of GFP^+^ cells to the wild-type littermates (Additional file 5: Figure S2b). When we tracked *Tet2*^–/–^GFP^+^ precursor division in culture, we found that the ratio of symmetric renewal to symmetric differentiation division was 1:2.5 for *Tet2* deleted precursors (Fig. 4c). This is dramatically different from the wild-type precursors whose ratio is 7:1 for symmetric renewal versus symmetric differentiation (Fig. 4a), suggesting that *Tet2* null HSCs/HPCs underwent more differentiation than wild-type precursors in our culture system. Of note, we found that the fluorescence intensity of the dividing cells of *Tet2* knockout was very low in comparison to wild-type littermates (Additional file 6: Figure S3a), suggesting that they are mostly progenitors. Intriguingly, co-culturing *Tet2* null hematopoietic precursors with OP9 stromal cells reversed the ratio to 3:1 for symmetric renewal versus symmetric differentiation (Fig. 4d), suggesting that OP9 stromal cells promote the renewal of *Tet2* deleted HSC/HPCs. Furthermore, treating *Tet2* deleted precursors with the hypomethylating agent 5-aza-2′-deoxycytidine (DAC) rescued the defect, suggesting that DNA methylation plays an important role in controlling the fate of precursors (Fig. 4e).

### *Tet2*^*–/–*^;*FLT3*^ITD^ leukemia precursors primarily underwent symmetric renewal divisions ex vivo

**Fig. 5:**
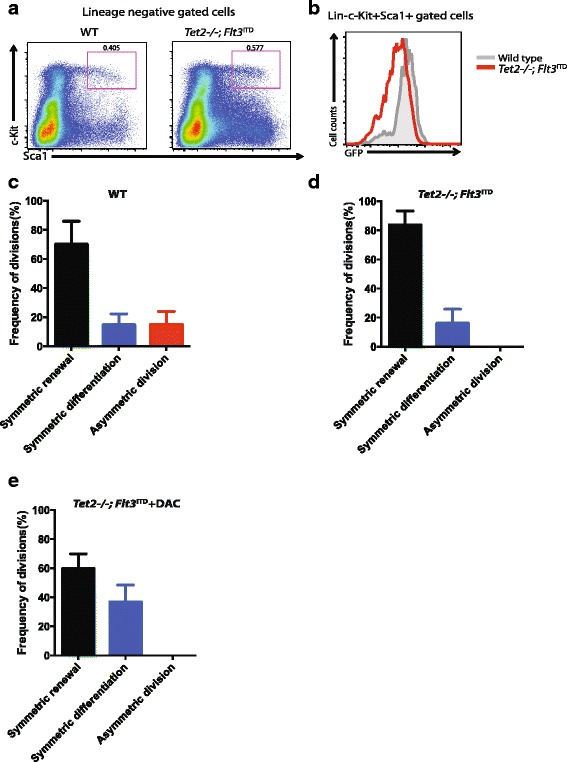
*Tet2*^*−/−*^;*Flt3*^ITD^ hematopoietic precursors underwent primarily symmetric renewal ex vivo*.* **a** Representative data from FACS analysis of wild-type (WT) Evi1-GFP and *Tet2*^*−/−*^;*Flt3*^ITD^;Evi1-GFP HSCs. The cells were stained with antibodies to lineage, Sca1, and c-Kit markers. The lineage negative population was gated first. Numbers indicate percent cells within Lin-c-Kit^+^Sca1^+^ gates. **b** Representative FACS data from GFP^+^ population from wild-type and *Tet2*^*−/−*^;*Flt3*^ITD^;Evi1-GFP mouse. The lineage, Sca1, and c-Kit markers were stained and gated first, then the GFP^+^ population from Lin-Sca1^+^c-Kit^+^ was compared with wild-type Evi1-GFP and *Tet2*^*−/−*^;*Flt3*^ITD^;Evi1-GFP mouse. **c** The frequency of symmetric self-renewal, symmetric differentiation, and asymmetric division of GFP^+^ from wild-type Evi1-GFP mouse bone marrow is shown. The data are a summary from three independent experiments. Error bars represent standard error of the mean (SEM). **d** The frequency of symmetric self-renewal, symmetric differentiation, and asymmetric division of GFP^+^ cells from *Tet2*^*−/−*^;*Flt3*^ITD^;Evi1-GFP compound mouse is shown. The data are a summary from three independent experiments. Error bars represent SEM. **e** The frequency of symmetric self-renewal, symmetric differentiation, and asymmetric division of 500 nM 5-aza-2′-deoxycytidine (DAC) treated GFP^+^ cells from *Tet2*^*−/−*^;*Flt3*^ITD^;Evi1-GFP compound mouse is shown. The data are a summary from three independent experiments. Error bars represent SEM

In a recent study, Shih et al. elegantly demonstrated that *Tet2*^−/−^ and *Flt3*^ITD^ alleles cooperate to induce AML in a mouse model. The hematopoietic precursors from this AML model have a unique transcriptional and epigenetic profiling signature [26]. To explore whether AML precursors have an aberrant cell division pattern, we crossed *Tet2*^−/−^;*Flt3*^ITD^ mouse to Evi1-GFP mouse, harvested GFP^+^ cells from *Tet2*^−/−^;*Flt3*^ITD^;Evi1-GFP mouse bone marrow and examined the phenotypic HSCs by flow cytometry. Furthermore, we also investigated the dividing pattern of *Tet2*^−/−^;*Flt3*^ITD^;Evi1-GFP hematopoietic precursors ex vivo by time-lapse microscopy. First, we found that the *Tet2*^−/−^;*Flt3*^ITD^;Evi1-GFP mouse has a modest increase in the percentage of bone marrow HSCs, whereas the overall GFP^+^ intensity was slightly reduced compared with wild-type littermates (Fig. 5a, b). Second, we observed that *Tet2*^−/−^;*Flt3*^ITD^;Evi1-GFP HSC/HPCs underwent more symmetric renewal than the control (Fig. 5c, d), consistent with the concept that oncogenes can subvert the balance of symmetric and asymmetric division of precursors [14, 27]. Being different from *Tet2* null precursors, we found that the fluorescence intensity of the dividing *Tet2*^−/−^;*Flt3*^ITD^ precursors was comparable to wild-type littermates (Additional file 6: Figure S3b). Finally, we also assessed the effect of DAC, an inhibitor of DNA methyltransferase, on *Tet2*^−/−^;*Flt3*^ITD^ precursor division. Our data showed that 500 nM DAC treatment significantly reduced the frequency of precursors dividing (from 23.4% to 6.0%) ex vivo. Moreover, DAC treatment significantly altered the dividing pattern of precursors and reduced symmetric renewal, whereas it promoted the differentiation of precursors (Fig. 5e).

Taken together, our studies have exploited a new reporter system for tracking division of hematopoietic precursors in real time and demonstrated that the balance of symmetric and asymmetric division can be influenced by extrinsic and intrinsic cues. Furthermore, we showed that leukemic precursors underwent more symmetric renewal divisions, suggesting that the aberrant symmetry of precursors is a feature of transformed cells and might be a target for leukemia therapy.

## Discussion

Hematopoietic stem cell transplantation is a mainstay of therapy for hematopoietic malignancies and a variety of hereditary disorders. Understanding the regulatory mechanisms of HSC self-renewal and differentiation is important for both basic stem cell biology and improving the quality of stem cell transplantation in clinical settings [34]. To study the signaling regulation of HSC/HPC ex vivo, a faithful HSC-specific reporter would be very instrumental to trace HSC activity in real time in culture. In this study, we exploited a novel reporter of hematopoietic precursors, Evi1-GFP, to track the division of hematopoietic precursors in culture. Consistent with a previous report [7], we demonstrated that Evi1 is expressed exclusively in the HSC population in the bone marrow and is indeed a faithful reporter of HSC activity in culture. One major advantage of the Evi1-GFP reporter system is that GFP knock-in does not affect the function of the EVI1, which plays a key role in hematopoietic precursors [5–7]. With this novel reporter, we found that both cytokine and growth factor combinations as well as stromal cells direct the division pattern of HSCs ex vivo. Deletion of a critical epigenetic regulator, TET2, in HSCs changed the division pattern of HSCs in culture. Our novel HSC reporter system provides a unique tool for investigating the regulatory mechanisms of HSC self-renewal and differentiation in culture. The knowledge learned from this study might contribute to the development of a protocol for clinically applicable human HSC expansion in the future.

Using a transgenic Notch reporter (TNR) mouse line, in which GFP fluorescence indicates the status of Notch signaling, Wu et al. [14] demonstrated that hematopoietic precursor cells can undergo both asymmetric and symmetric divisions. Moreover, the stromal cells on which HSCs have been placed also affect the pattern of division. Furthermore, it was nicely demonstrated that oncogenes can also influence the balance of symmetric and asymmetric division [14]. This conclusion is consistent with the idea that symmetric and asymmetric cell division is an essential mechanism of balancing self-renewal and differentiation of HSCs. The major issue with this study is that it is quite controversial whether this notch reporter is a specific reporter of HSC activity in bone marrow [35, 36].

How stem cells divide has been extensively studied. The best example of symmetric and asymmetric division is the dividing of precursor cells in invertebrates. In the Drosophila germline, when a stem cell divides asymmetrically it is always the cell close to the “hub cell” that remains a stem cell while the other differentiates. It is suggested that a signal from the hub is critical to determine the fate of the dividing cells, for example the unpaired ligand [37, 38]. Another example is in the Drosophila nervous system; the neuroblast divides along its apical basal axis such that the apical daughter remains a neuroblast and the basal daughter becomes a ganglion mother cell that generates differentiated progeny [39]. It also has been reported that two stem cells from the *Caenorhabditis elegans* germline can divide to become several thousand germ cells through a series of symmetric divisions [40]. It has been documented that there is a similar division pattern in most mammalian systems, including the hematopoiesis system. Asymmetric fates of the progeny of mammalian hematopoietic precursor cells have been described previously in a number of elegant studies using paired daughter cell analyses and microscopic time-lapse imaging [41–43]. It has also been demonstrated that the frequency of these asymmetric fate decisions can be influenced by extrinsic signals provided by the type of stromal or cytokines used in culture [14, 44]. Our data are very consistent with the literature that mammalian stem cells can undergo asymmetric and symmetric division. Extrinsic signals, e.g., cytokines and growth factors, can control the balance of symmetric and asymmetric division. Recent studies reported ex vivo expansion of hematopoietic stem and progenitor cells using cytokine cocktails combined with an array of factors, including Musashi-2, Pyrimidoindole derivatives (UM171), aryl hydrocarbon receptor antagonists (SR1), Wnt activators, Notch ligands, angiopoietin-like proteins, prostaglandin E2, pleiotrophin, and GSK-3 inhibitor plus insulin [8, 12, 45–48]. It would be very interesting to examine the effect of these factors on HSC division ex vivo in the future.

Wild-type OP9 stromal cells can partially reverse the division defect of *Tet2* knockout (KO) precursors, suggesting that the *Tet2* KO niche also contributes to the hematopoiesis phenotype of *Tet2* KO mouse. It is well established that the stromal niche regulates quiescence, multipotentiality, and self-renewal of HSCs in vivo [49, 50]. It is also known that the hematopoiesis-specific Cre Mx1-Cre is expressed in niche stromal cells of bone marrow [51]. Thus, the in vivo *Tet2* KO phenotypes might be the combination effect of *Tet2* deletion in precursors and stromal niche cells.

Previous studies demonstrated that deletion of *Tet2* in HSCs leads to an increased primitive compartment including both stem and progenitor cells in vivo [23–25]. Our data showed that *Tet2* null HSCs underwent more symmetric differentiation than wild-type HSCs ex vivo, suggesting that loss of *Tet2* induces differentiation of hematopoietic precursors in culture. Those dividing *Tet2* null precursors in general displayed low GFP intensity. Given that GFP intensity is correlated with “stemness” of precursors, it is very likely that those dividing cells are mostly progenitors. These data are consistent with in vivo studies showing that *Tet2* deletion expands both hematopoietic stem cells and progenitor compartments [23–25]. Wild-type OP9 can reverse the phenotype of *Tet2* null precursors, indicating that microenvironment contributes to the aberrant dividing pattern of *Tet2* null precursors in culture. Importantly, we also demonstrated that the DNA methyltransferase inhibitor decitabine (DAC) rescued the division defect of *Tet2* knockout, suggesting the DNA methylation is responsible for causing the division defect of precursors. Lastly, we showed that *Tet2*^−/−^;*Flt3*^ITD^ HSCs/HPCs underwent more symmetric renewal division, which is consistent with the idea that some oncogenes influence the balance of symmetric and asymmetric division [14, 27]. This finding provides a rationale for targeting the abnormal cell division of leukemic precursors as a new therapeutic approach to leukemia.

There are several limitations to the current study, the most important one being the specificity of the Evi1-GFP reporter. In our transplantation experiment, only one-third of the mice were successfully engrafted upon injecting 100 Evi1-GFP^+^ cells. The engraftment ability of Evi1-GFP^+^ cells is weaker in comparison to sorted HSCs, indicating that not all GFP^+^ cells are HSCs in Evi1-GFP mouse. Consistently, we showed that GFP intensity is correlated with “stemness” of precursors. Although it is still important to investigate the behaviors of all hematopoietic precursors in culture, it would be also critical to isolate bona fide HSCs and monitor their dividing behavior in our system in future. The second limitation is that we only tracked precursors dividing for 24 h in this study. It is known that the time until the first division for HSCs is on average 45 h ex vivo [52]. In future, the long-term time-lapse tracking of precursors will provide more valuable information about self-renewal and differentiation features of HSCs in culture.

## Conclusions

In summary, we demonstrated that Evi1-GFP mouse is a faithful reporter of hematopoietic precursors ex vivo. A cytokine and growth factor (STIF) combination promotes symmetric renewal of precursors in culture whereas OP9 stromal cells balance the renewal and differentiation of precursors. We found that *Tet2* deletion leads to increased symmetric differentiation of precursors ex vivo*. Tet2*^*–/–*^;*FLT3*^ITD^ AML precursors primarily underwent symmetric renewal divisions in culture. Importantly, we found that inhibiting DNA methylation can reverse the aberrant division phenotypes of *Tet2*^*–/–*^ and *Tet2*^*–/–*^;*FLT3*^ITD^ precursors, suggesting that abnormal DNA methylation plays an important role in controlling (pre-)leukemic precursor fate decision ex vivo.

## Additional files

Additional file 1: Movie S1. GFP^+^ precursors underwent symmetric renewal ex vivo*.* The representative time-lapse movie of GFP^+^ precursors that underwent symmetric renewal during 24 h in culture. (AVI 1022 kb)

Additional file 2: Movie S2. GFP^+^ precursors underwent symmetric differentiation ex vivo*.* The representative time-lapse movie of GFP+ precursors that underwent symmetric differentiation during 24 h in culture. (AVI 1118 kb)

Additional file 3: Movie S3. GFP^+^ precursors underwent asymmetric division ex vivo*.* The representative time-lapse movie of GFP^+^ precursors that underwent asymmetric division during 24 h in culture. (AVI 798 kb)

Additional file 4: Figure S1. OP9 stromal cells contribute to the fate decision of precursors ex vivo*.* (a) The frequency of symmetric self-renewal, symmetric differentiation, and asymmetric division of GFP^+^ precursors from wild-type mouse treated with OP9 supernatant during 24 h is shown. The data are a summary from three independent experiments. Error bars represent standard error of the mean (SEM). (b) The frequency of symmetric self-renewal, symmetric differentiation, and asymmetric division of GFP^+^ precursors from wild-type mouse seeded on irradiated OP9 cells during 24 h is shown. The data are a summary from three independent experiments. Error bars represent SEM. (PDF 420 kb)

Additional file 5: Figure S2. *Tet2* loss leads to increased hematopoietic stem cell in mouse bone marrow. (a) The representative data of FACS analysis of wild-type and *Tet2* knockout HSCs. The cells were stained with antibodies to lineage, Sca1, and c-Kit markers. The lineage negative population was gated first. Numbers indicate percent cells within Lin-c-Kit^+^Sca1^+^ gates. (b) The representative FACS data of GFP^+^ population from wild-type and *Tet2*^−/−^ mouse. The lineage, Sca1, and c-Kit markers were stained and gated first. The GFP^+^ population from Lin-Sca1^+^c-Kit^+^ was compared between wild-type and *Tet2*^−/−^ mouse. (PDF 642 kb)

Additional file 6: Figure S3. The fluorescence intensity of dividing *Tet2*^–/–^ and *Tet2*^−/−^;*Flt3*^ITD^ dividing HSCs. (a) The GFP pixel intensity unit (PIU) of *Tet2*^–/–^ dividing HSCs was compared with wild-type control. (b) The GFP PIU of *Tet2*^–/–^;*Flt3*^ITD^ dividing HSCs was compared with wild-type control. (PDF 552 kb)

## Abbreviations

AML: Acute myeloid leukemia
DAC: 5-Aza-2′-deoxycytidine
DMEM: Dulbecco’s modified Eagle’s medium
DNMT: DNA methyltransferase enzyme
Evi1: Ecotropic viral integration site 1
FGF: Fibroblast growth factor
FLT3-L: FMS-like tyrosine kinase-3 ligand
GFP: Green fluorescent protein
HSC: Hematopoietic stem cell
HSPC: Hematopoietic stem/progenitor cells
IGF: Insulin-like growth factor
ITD: Internal tandem duplication
KO: Knockout
LSC: Leukemia stem cell
LSK: Lin- Sca1^+^c-kit^+^ cells
PE: Phycoerythrin
PIU: Pixel intensity unit
SCF: Stem cell factor
STIF: SCF + TPO + IGF-2 + FGF-1
TET: Ten-eleven translocation
TPO: Thrombopoietin
