# Supplementary material for: Tracking hematopoietic precursor division ex vivo in real time
Source: Stem Cell Res Ther. 2018 Jan 23;9:16. doi: 10.1186/s13287-017-0767-z (PMC5781326; doi:10.1186/s13287-017-0767-z)
Supplement: Supplementary file 4 — OP9 stromal cells contribute to the fate decision of precursors ex vivo. (a) The frequency of symmetric self-renewal, symmetric differentiation, and asymmetric division of GFP+ precursors from wild-type mouse treated with OP9 supernatant during 24 h is shown. The data are a summary from three independent experiments. Error bars represent standard error of the mean (SEM). (b) The frequency of symmetric self-renewal, symmetric differentiation, and asymmetric division of GFP+ precursors from wild-type mouse seeded on irradiated OP9 cells during 24 h is shown. The data are a summary from three independent experiments. Error bars represent SEM. (PDF 420 kb) [file 13287_2017_767_MOESM4_ESM.pdf]

Tracking Hematopoietic Precursor Division *ex vivo* in Real Time

Running title: Tracking Division of Hematopoietic Precursors in Culture

Yuchen Wang, Hong Tian, Wenzhi Cai, Zhaorui Lian, Dheeraj Bhavanasi, Chao Wu, Tomohiko Sato, Mineo Kurokawa, De-Pei Wu, Li Fu, Hong Wang, Hao Shen, Dong Liang, Jian Huang

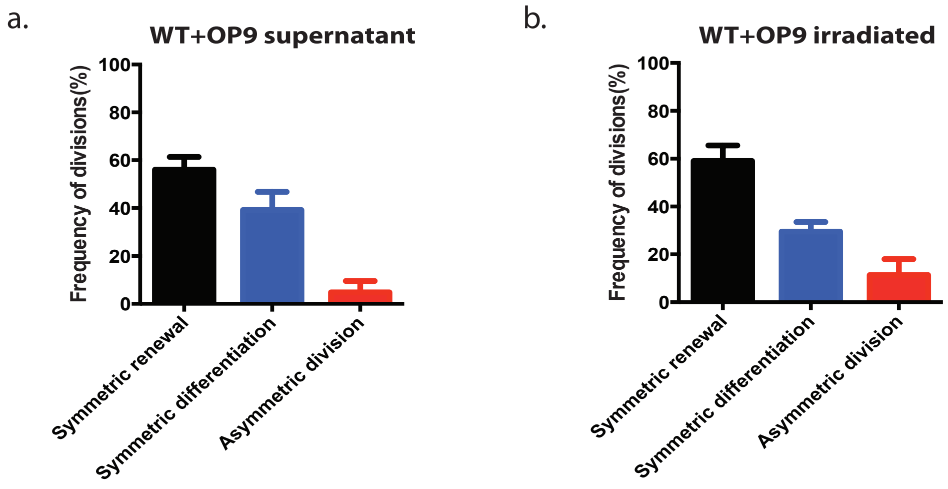

Figure S1. OP9 stromal cells contribute to the fate decision of precursors *ex vivo*
(a) The frequency of symmetric self-renewal, symmetric differentiation and asymmetric division of GFP+ precursors from wild type mouse treated with OP9 supernatant during 24 hours is shown.The data was a summary from three independent experiments. Error bars represent standard standard error of the mean (SEM).
(b) The frequency of symmetric self-renewal, symmetric differentiation and asymmetric division of GFP+ precursors from wild type mouse seeded on irradiated OP9 cells during 24 hours is shown.The data was a summary from three independent experiments. Error bars represent standard standard error of the mean (SEM).
