# Supplementary material for: Tracking hematopoietic precursor division ex vivo in real time
Source: Stem Cell Res Ther. 2018 Jan 23;9:16. doi: 10.1186/s13287-017-0767-z (PMC5781326; doi:10.1186/s13287-017-0767-z)
Supplement: Supplementary file 5 — Tet2 loss leads to increased hematopoietic stem cell in mouse bone marrow. (a) The representative data of FACS analysis of wild-type and Tet2 knockout HSCs. The cells were stained with antibodies to lineage, Sca1, and c-Kit markers. The lineage negative population was gated first. Numbers indicate percent cells within Lin-c-Kit+Sca1+ gates. (b) The representative FACS data of GFP+ population from wild-type and Tet2−/− mouse. The lineage, Sca1, and c-Kit markers were stained and gated first. The GFP+ population from Lin-Sca1+c-Kit+ was compared between wild-type and Tet2−/− mouse. (PDF 642 kb) [file 13287_2017_767_MOESM5_ESM.pdf]

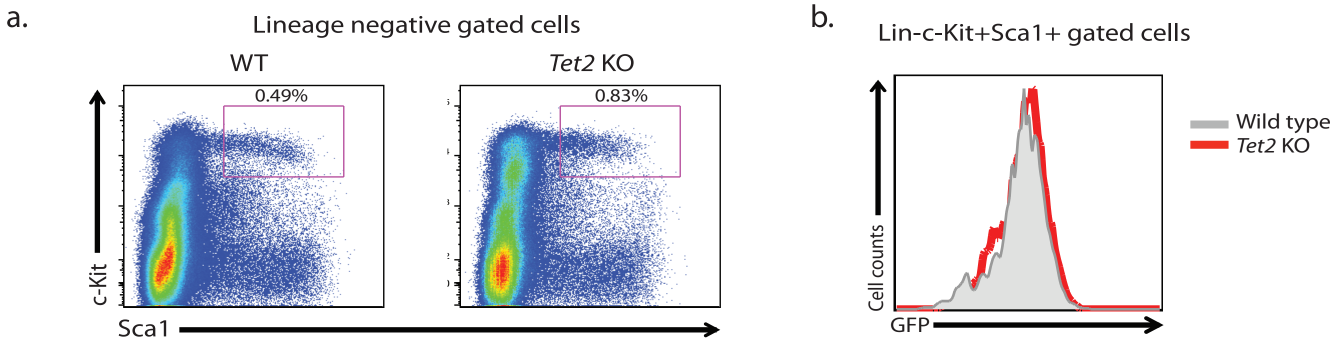

Figure S2. ***Tet2 loss leads to increased hematopoietic stem cell in mouse bone marrow***
(a) The representative data of FACS analysis of wild type and *Tet2* knockout HSCs. The cells were stained with antibodies to lineage, Sca1 and c-Kit markers. The lineage negative population was gated first. Numbers indicate percent cells within Lin-c-Kit+Sca1+ gates.
(b) The representative FACS data of GFP+ population from wild type and *Tet2*–/– mouse. The lineage, Sca1 and c-Kit markers were stained and gated first, then The GFP+ population from Lin-Sca1+c-Kit+ was compared between wild type and *Tet2*–/– mouse.
