# Supplementary material for: Tracking hematopoietic precursor division ex vivo in real time
Source: Stem Cell Res Ther. 2018 Jan 23;9:16. doi: 10.1186/s13287-017-0767-z (PMC5781326; doi:10.1186/s13287-017-0767-z)
Supplement: Supplementary file 6 — The fluorescence intensity of dividing Tet2–/– and Tet2−/−;Flt3ITD dividing HSCs. (a) The GFP pixel intensity unit (PIU) of Tet2–/– dividing HSCs was compared with wild-type control. (b) The GFP PIU of Tet2–/–;Flt3ITD dividing HSCs was compared with wild-type control. (PDF 552 kb) [file 13287_2017_767_MOESM6_ESM.pdf]

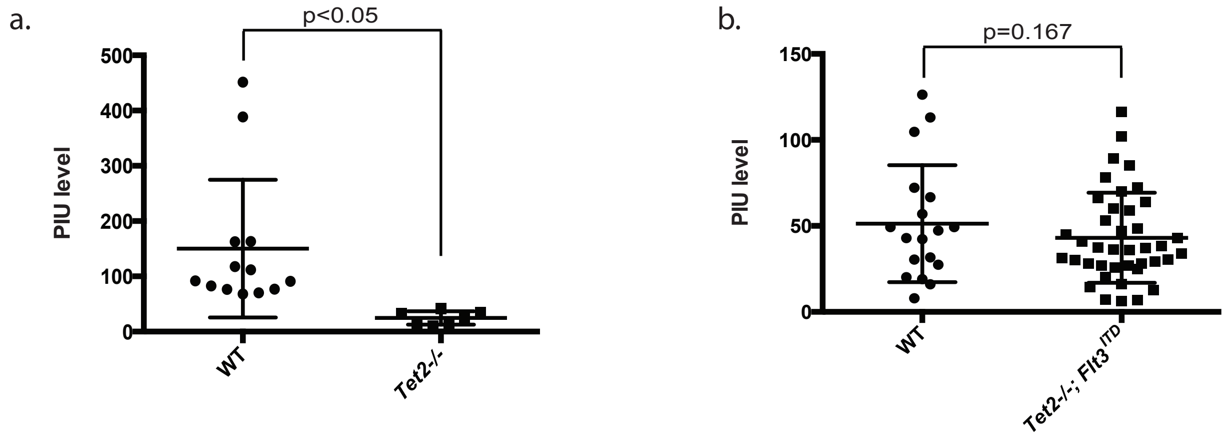

Figure S3. The fluorescence intensity of dividing *Tet2 -/-* and *Tet2 -/-; Flt3*^{ITD} dividing HSCs.
(a) The GFP pixel intensity unit (PIU) of *Tet2-/-* dividing HSC was compared to wild type control.
(b) The GFP pixel intensity unit (PIU) of *Tet2-/-*; *Flt3*^{ITD} dividing HSC was compared to wild type control.
